# Comparative Mitogenomics of *Channa pyrophthalmus* Unveils Orogeny-Driven Speciation and Lineage-Specific Adaptive Evolution in Snakeheads

**DOI:** 10.3390/ani16030467

**Published:** 2026-02-02

**Authors:** Qing Luo, Jiafeng Liu, Jiajun Liu, Mi Ou, Shuzhan Fei, Haiyang Liu, Xincheng Zhang, Jian Zhao

**Affiliations:** 1 Pearl River Fisheries Research Institute, Chinese Academy of Fishery Sciences, Guangzhou 510380, China; luoqing@prfri.ac.cn (Q.L.); om1990@prfri.ac.cn (M.O.); feisz@prfri.ac.cn (S.F.); hyliu@prfri.ac.cn (H.L.); zhangxc@prfri.ac.cn (X.Z.); 2 Key Laboratory of Tropical & Subtropical Fishery Resources Utilization & Cultivation, Ministry of Agriculture, Guangzhou 510380, China; 3 College of Fisheries and Life Science, Shanghai Ocean University, Shanghai 201306, China; 4 School of Marine Science and Fisheries, Jiangsu Ocean University, Lianyungang 222005, China

**Keywords:** mitogenome, *Channa pyrophthalmus*, Indo-Burman Ranges, divergence time, adaptive evolution, body size

## Abstract

Snakeheads of the genus *Channa* display remarkable diversity in body size and ecology, yet evolutionary relationships within several species complexes remain unresolved. *Channa pyrophthalmus*, a recently described dwarf species endemic to Myanmar, represents a key lineage for investigating allopatric diversification in freshwater fishes. Here, we report the first complete mitochondrial genome of *C. pyrophthalmus* and perform comparative mitogenomic analyses across 18 *Channa* species. Phylogenetic analyses based on mitochondrial protein-coding genes robustly place *C. pyrophthalmus* as the sister lineage to *C. gachua* sensu stricto, supporting recent taxonomic revisions. While mitochondrial coding regions are highly conserved across the genus, the control region exhibits pronounced lineage-specific structural variation driven by tandem repeats. Divergence-time estimation dates the split between *C. pyrophthalmus* and *C. gachua* to ∼7.1 Ma (Late Miocene), a timeline congruent with the Late Miocene accelerated uplift of the Indo-Burman Ranges. Selection analyses reveal contrasting evolutionary regimes: pervasive purifying selection characterizes the dwarf lineage, whereas episodic positive selection on *ND5* marks the ancestral lineage of giant snakeheads, indicating adaptive shifts in mitochondrial bioenergetics. Additionally, accelerated evolution in *ATP8* was detected in rheophilic lineages. Together, these findings link geological vicariance and mitochondrial metabolic evolution to body size diversification in *Channa*.

## Simple Summary

Snakeheads are iconic freshwater predatory fishes in Asia, but their classification is often difficult due to similar appearances. Recently, a new species, the Fire and Ice Snakehead (*Channa pyrophthalmus*), was discovered in the Tenasserim Region of Myanmar. In this study, we decoded its complete mitochondrial genome to understand its evolutionary history. Our analysis confirms that this species is genetically distinct and split from its closest relatives approximately 7 million years ago, likely isolated by ancient mountains. Interestingly, unlike the widespread Striped Snakehead (*Channa striata*) which evolved rapidly to adapt to diverse environments, the Fire and Ice Snakehead shows a conservative evolutionary strategy, maintaining a stable genome suited to its specific mountain stream habitat. This study highlights the importance of the Tenasserim region as a biodiversity shelter and provides genetic tools for identifying and protecting this unique fish.

## 1. Introduction

Snakeheads, belonging to the family Channidae, are freshwater teleosts easily distinguished by their elongated, cylindrical bodies, formidable dentition, and the possession of a suprabranchial organ facilitating air-breathing [1,2]. Distributed extensively across Africa and Asia, these apex predators hold substantial economic value, serving as key resources for both aquaculture and the ornamental fish trade [3]. Among the channids, the Asian genus *Channa* represents the most speciose and morphologically diverse lineage, displaying extraordinary variation in life history traits—ranging from dwarf species (<25 cm) inhabiting hill streams to riverine giants exceeding a meter in length [3].

Historically, the taxonomy of *Channa* has proven challenging. Morphological conservatism and high phenotypic plasticity have obscured species boundaries, resulting in several unresolved species complexes, notably surrounding *Channa striata*, *Channa marulius*, and *Channa gachua* [4,5]. The *C. gachua* group is particularly problematic, representing a widespread and taxonomically intricate lineage centered on the Eastern Himalaya and the broader Indo-Burma Biodiversity Hotspot [3]. However, recent integrative taxonomic efforts have begun to disentangle this diversity, revealing numerous endemic species previously subsumed under broad nominal taxa [6,7]. One such species is *Channa pyrophthalmus*, a recently described dwarf species endemic to the Tanintharyi Region of Myanmar—a critical center of endemism within the hotspot. Notably, due to its striking bright orange suborbital patch and steel-blue lips, this species has recently gained economic importance in the international ornamental fish trade, where it is marketed under the name “Fire and Ice Snakehead” [7]. Geographically isolated west of the Tenasserim barrier, *C. pyrophthalmus* occupies a restricted range distinct from its widespread congeners, making it an excellent model for investigating allopatric speciation and local adaptation [7].

Mitochondrial genomes (mitogenomes) offer robust, high-resolution markers for reconstructing phylogenies and exploring molecular evolution, given their maternal inheritance, lack of recombination, and rapid evolutionary rates [8,9,10]. While single-locus markers like *COX1* and *CYTB* are standard for barcoding [5,11], they often provide insufficient resolution for resolving deep nodes or detecting complex signals such as adaptive selection. Recent efforts have successfully characterized complete mitogenomes for various *Channa* species, including *Channa argus* [12,13,14], *Channa maculata* [13,14,15], *C. striata* [16], and the Gachua group members like *Channa stewartii* and *Channa pulchra* [17]. Despite this progress, genomic data for *C. pyrophthalmus* remains absent, leaving gaps in our understanding of how phenotypic traits and biogeographical events in the Indo-Burman Ranges (IBR) have shaped this distinct lineage.

Here, we report the complete mitochondrial genome of *C. pyrophthalmus* obtained via next-generation sequencing. Through a comprehensive analysis against 17 other *Channa* mitogenomes, we aim to elucidate genomic architecture and structural variations, with specific attention to the control region. Concurrently, we reconstruct a robust time-calibrated phylogeny to evaluate biogeographical hypotheses concerning the uplift of the Indo-Burman Ranges. Finally, by investigating selective pressures on protein-coding genes, we seek to uncover the molecular basis of adaptation, offering new insights into the mitochondrial bioenergetic constraints underlying body size evolution in freshwater teleosts.

## 2. Materials and Methods

### 2.1. Sample Collection and Molecular Identification

A specimen of *C. pyrophthalmus* was purchased from a licensed commercial ornamental fish vendor in the Baiyi Ornamental Fish Market (Guangzhou, China). This species is known to be imported from Myanmar for the aquarium trade. Species identification was confirmed based on diagnostic morphological characters following the original description [7] and further validated by *COX1* sequence comparison with reference sequences from type material (Figure S1). The specimen has been deposited in the China-ASEAN Fisheries Resources Database (Voucher No. Cpy250302003). Genomic DNA extraction was carried out using the E.Z.N.A. Tissue DNA Kit (Omega Bio-tek, Norcross, GA, USA) according to the manufacturer’s instructions, with quality and concentration quantified via the TBS 380 fluorometer (Invitrogen, Carlsbad, CA, USA) and Pico Green Assay (Life Technologies, Carlsbad, CA, USA). We constructed the sequencing library using the Illumina TruSeq^TM^ Nano DNA Sample Prep Kit (Illumina, San Diego, CA, USA). The experimental protocol was approved by the Animal Ethics Committee of the Pearl River Fisheries Research Institute, Chinese Academy of Fishery Sciences (LAEC-PRFRI-2024-12-04).

### 2.2. Mitogenome Sequencing and Assembly

Genomic sequencing was conducted on the Illumina NovaSeq 6000 platform (Illumina, San Diego, CA, USA). We processed raw reads with Trimmomatic v0.39 to eliminate adaptors and low-quality bases, followed by de novo assembly using GetOrganelle v1.7.5 [18].

Preliminary annotation was performed via the MITOS2 web server, utilizing the vertebrate mitochondrial code. To ensure high accuracy, we imported the sequences into Geneious Prime v2025.1.3 (Biomatters Ltd., Auckland, New Zealand) for manual curation, refining start and stop codons through comparative alignment with orthologous sequences of other *Channa* species. The final circular map was visualized using OGDRAW v1.3.1 [19]. Additionally, tRNA secondary structures were predicted using tRNAscan-SE v2.0 [20] and rendered via the Forna web server (http://rna.tbi.univie.ac.at/forna/, accessed on 23 November 2025).

### 2.3. Comparative Genomic Analysis

For comparative purposes, we compiled a dataset comprising 17 *Channa* mitogenomes and one outgroup *Parachanna insignis* from GenBank (details in Table S1) [16,17,21]. We assessed nucleotide diversity (π) via sliding window analysis in DnaSP v6 [22], setting a window size of 100 bp with a 25 bp step. Synonymous (Ks) and non-synonymous (Ka) substitution rates were also estimated using DnaSP v6, with Ka/Ks ratios calculated to infer selection pressures on each protein-coding gene (PCG). Evolutionary metrics, including pairwise genetic distances (Kimura-2-parameter, K2P) and AT/GC-skewness, were computed using PhyloSuite v2 [23]. We also calculated Relative Synonymous Codon Usage (RSCU) to evaluate codon usage bias across the dataset. The structural organization of the control region was analyzed to identify conserved motifs (TAS, CSB) and tandem repeats. Tandem repeats were detected using the Tandem Repeats Finder (TRF) server (https://tandem.bu.edu/trf/trf.html, accessed on 10 December 2025) and manually verified.

### 2.4. Phylogenetic Reconstruction and Divergence Time Estimation

Phylogenetic inferences were based on a concatenated dataset of 13 protein-coding genes (PCGs). Sequences were aligned using MAFFT v7 [24] and subsequently trimmed with Gblocks. We assessed substitution saturation using DAMBE7 [25]. Phylogenetic tree reconstruction employed both Maximum Likelihood (ML) and Bayesian Inference (BI) frameworks. ML analysis was conducted in IQ-TREE v3 [26] using the best-fit model (GTR+F+R4) determined by ModelFinder [27], with robustness assessed via 5000 bootstrap replicates. BI analysis was performed using MrBayes v3.2 [28], running for 2 million generations (sampling every 1000 generations) and discarding the initial 25% as burn-in. The resulting topologies were visualized and refined in iTOL (https://itol.embl.de/, accessed on 22 December 2025) [29].

We estimated divergence times using BEAST v2.7 [30]. The dataset was analyzed using the SRD06 partitioning strategy [31], dividing the nucleotide alignment into two partitions: combined 1st and 2nd codon positions, and the 3rd codon position. Substitution models (GTR+G) and rate heterogeneity parameters were unlinked across partitions, while the clock model and tree topology were linked. The analysis applied a Yule speciation prior and an uncorrelated relaxed clock (log-normal). To calibrate the tree, we used a secondary calibration point at the Channidae crown node based on the time estimates from Calibration Scheme 1 of Rüber et al. [3], which incorporates the fossil *Parachanna fayumensis*. Accordingly, we set a Log-Normal prior (offset = 33.0 Ma, log mean = 0.1, log SD = 0.8). We constrained the monophyly of the genus *Channa*. The MCMC chain ran for 50 million generations, with convergence confirmed in Tracer v1.7 [32] (ESS > 200). A maximum clade credibility (MCC) tree was generated using TreeAnnotator after a 10% burn-in.

### 2.5. Selection Pressure and Structural Analysis

To screen for signals of adaptive evolution, we employed the adaptive Branch-Site Random Effects Likelihood (aBSREL) method within HyPhy (https://www.hyphy.org/, accessed on 10 December 2025) [33]. Statistical significance was determined using the Likelihood Ratio Test (LRT) at *p* < 0.05. Where significant positive selection was detected, we mapped specific selected sites onto the 3D protein structure predicted by AlphaFold3 (https://alphafoldserver.com/, accessed on 10 December 2025) [34] and visualized them using PyMOL v3.1.0.

## 3. Results

### 3.1. Genome Organization and Characterization

**Figure 1:**
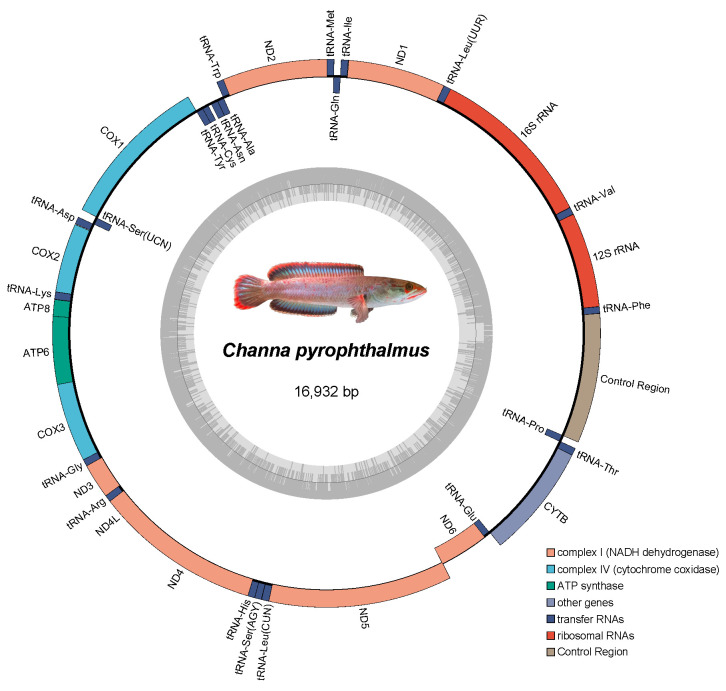
Circular map of the mitochondrial genome of *C. pyrophthalmus*. The genome is 16,932 bp in length. Genes shown on the outer circle are transcribed from the heavy strand (H-strand), while those on the inner circle are transcribed from the light strand (L-strand). The genes are color-coded based on their functional classification (see bottom right legend). The inner grey ring represents the GC content graph. The central photograph shows the specimen of *C. pyrophthalmus*.

The newly sequenced mitochondrial genome of *C. pyrophthalmus* forms a closed circular molecule of 16,932 bp in length (GenBank Accession No. PX764270). It exhibits the typical vertebrate mitochondrial gene arrangement, comprising 13 protein-coding genes (PCGs), 22 tRNAs, 2 rRNAs, and a control region (Table 1, Figure 1). Nucleotide composition is biased toward A+T (55.4%). Regarding start codons, most PCGs utilize standard ATG, with the exception of *COX1*, which initiates with GTG. Incomplete stop codons (T––) were observed in four genes. These are likely completed via post-transcriptional polyadenylation.

RSCU analysis highlighted Leucine as the most frequently utilized amino acid, contrasting with Cysteine, which was the least frequent (Figure S2). Consistent with the genome-wide A+T bias, codons ending in A or T were preferentially selected. Structural analysis of the 22 tRNAs showed that they range from 65 to 75 bp in length and fold into typical cloverleaf structures, with the notable exception of *tRNA-Ser(AGY)*, which lacks the dihydrouridine arm (Figure S3).

### 3.2. Phylogenetic Relationships and Divergence Times

**Figure 2:**
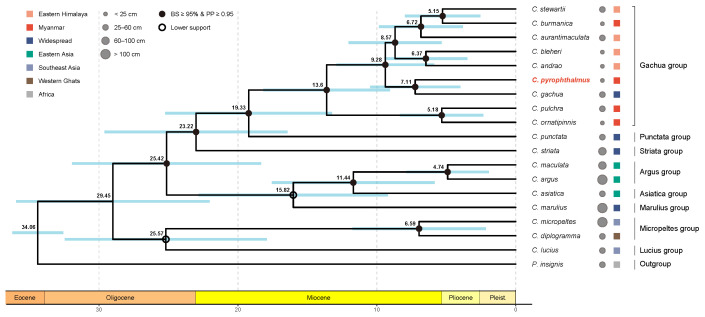
Phylogenetic relationships and divergence times of the genus *Channa*. The chronogram was inferred from the concatenated nucleotide sequences of 13 mitochondrial protein-coding genes using Bayesian inference (BEAST v2.7). The topology is congruent with the Maximum Likelihood (ML) analysis. (Left) Tree Topology and Dating: Solid black circles at nodes indicate high nodal support (ML bootstrap support ≥ 95% and Bayesian posterior probability ≥ 0.95); open circles indicate lower support. Numbers near nodes denote mean divergence times in million years (Ma). Blue bars represent 95% highest posterior density (HPD) intervals. The geological time scale is displayed at the bottom. *Parachanna insignis* was used as the outgroup. The newly described species, *C. pyrophthalmus*, is highlighted in red. (Right) Metadata Mapping: (1) Body Size: Grey circles indicate the maximum standard length (SL), categorized into four classes (<25 cm, 25–60 cm, 60–100 cm, and >100 cm), highlighting the correlation between phylogenetic position and body size (dwarf vs. giant lineages). (2) Distribution: Colored squares represent native geographic ranges: Orange, Eastern Himalaya; Red, Myanmar; Dark Blue, Widespread; Green, Eastern Asia; Steel Blue, Southeast Asia; Brown, Western Ghats; Grey, Africa. (3) Classification: Vertical brackets demarcate the major species groups within *Channa*.

Both ML and BI analyses yielded congruent topologies with robust nodal support across all major clades (Figure 2 and Figure S5). *C. pyrophthalmus* was unambiguously resolved as the sister taxon to *C. gachua*, a placement supported by maximal values (BS = 99, PP = 1.00), firmly embedding it within the Gachua group. The designation of major species groups within the genus follows the comprehensive phylogenetic framework established by Rüber et al. [3].

Our time-calibrated phylogeny (Figure 2) dates the origin of the family Channidae (the *Parachanna*–*Channa* split) to the Oligocene, approximately 29.45 Ma (95% HPD: 21.1–35.0 Ma). The radiation of the Gachua group appears to have initiated in the Middle Miocene (∼19.3 Ma). More specifically, the speciation event separating *C. pyrophthalmus* from its sister species *C. gachua* is estimated at 7.1 Ma (95% HPD: 4.0–10.5 Ma). This Late Miocene divergence coincides with the intensification or accelerated deformation phase of the Indo-Burman Ranges.

### 3.3. Comparative Genomic Landscape

**Figure 3:**
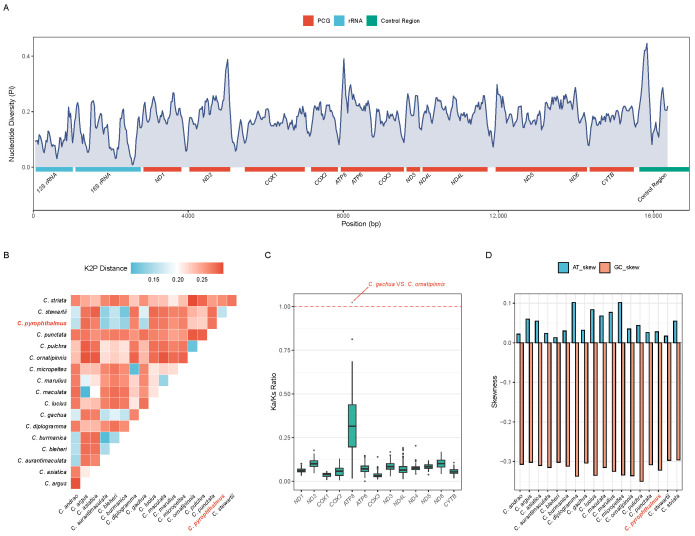
Comparative genomic analysis of *Channa* species based on mitochondrial genomes. (**A**) Nucleotide diversity (π) across the complete mitochondrial genomes calculated using a sliding window analysis. The line plot illustrates the distribution of π values, with the gene organization shown at the bottom. Functional regions are color-coded: red for PCGs, blue for rRNAs, and green for the control region. (**B**) Heatmap of pairwise genetic distances based on the K2P model. The lower triangular matrix represents the divergence between species pairs, with the color scale ranging from cyan (lower distance) to red (higher distance). The target species, *C. pyrophthalmus*, is highlighted in red bold. (**C**) Selection pressure (Ka/Ks) of the 13 mitochondrial PCGs. The boxplot summarizes the distribution of synonymous and non-synonymous substitution ratios across all species comparisons. The red dashed line at Ka/Ks = 1.0 indicates neutral evolution. While purifying selection (*ω* < 1) is dominant, a single outlier in *ATP8* exceeds 1.0, suggesting potential positive selection or relaxed functional constraints in specific lineages. (**D**) Nucleotide composition bias represented by AT-skew and GC-skew. Blue and orange bars denote AT-skew and GC-skew values, respectively, for each species. *C. pyrophthalmus* is highlighted in red bold to show its relative position in base composition bias compared to its congeners.

A comparative assessment of 18 *Channa* mitogenomes revealed distinct evolutionary heterogeneity (Figure 3). Sliding window analysis of nucleotide diversity (π) identified the control region, *ATP8*, and *ND2* as mutational hotspots, whereas *12S rRNA* exhibited high conservation (Figure 3A). In terms of genetic distance (K2P), *C. pyrophthalmus* is most divergent from *C. argus* and shows the closest affinity to other members of the Gachua group (Figure 3B).

Selection pressure analysis generally indicated pervasive purifying selection across the 13 PCGs, with median Ka/Ks ratios consistently falling below 1.0 (Figure 3C). *COX1* displayed the lowest ratio, underscoring its functional rigidity. However, an exception to this trend was observed in the *ATP8* gene: a specific pairwise comparison between *C. gachua* and *C. ornatipinnis* yielded a Ka/Ks ratio of 1.02. Given the short length of the *ATP8* gene, this elevated Ka/Ks ratio should be interpreted cautiously, as estimates for small genes are more sensitive to stochastic variation and lineage-specific effects. This represents the only instance across the entire mitogenome where the neutrality threshold was exceeded, suggesting accelerated evolution in this lineage. Additionally, strand-specific mutational bias was evident across all species, characterized by positive AT-skew and negative GC-skew in the majority of PCGs (Figure 3D).

## 4. Signatures of Adaptive Evolution

**Figure 4:**
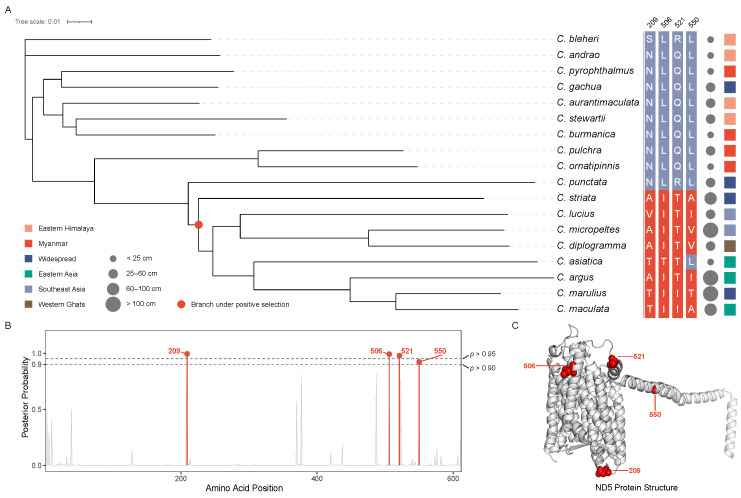
Evidence of episodic positive selection on the mitochondrial *ND5* gene in *Channa*. (**A**) Phylogenetic context of adaptive mutations. The tree topology illustrates the evolutionary relationships among *Channa* species. The red node indicates the ancestral branch (Node 5) where significant episodic positive selection was detected (aBSREL, *p* < 0.05). The heatmap to the right displays the amino acid residues at the four positively selected sites (209, 506, 521 and 550). Red boxes indicate derived residues, while grey/blue boxes represent the ancestral residues. Metadata columns indicate: (i) Body Size: Circle area corresponds to maximum standard length, highlighting the gigantism in the clade under positive selection. (ii) Native Distribution: Colored squares denote biogeographic origins (legend: Orange, Eastern Himalaya; Red, Myanmar; Dark Blue, Widespread; Green, Eastern Asia; Light Blue, Southeast Asia; Brown, Western Ghats). (**B**) Site-specific posterior probabilities. Manhattan plot showing the posterior probability of positive selection for each codon position in ND5. The four significant sites (209, 506, 521 and 550) exceed the high confidence thresholds (dashed lines at 0.90 and 0.95). (**C**) Structural mapping of adaptive sites. The predicted tertiary structure of the ND5 protein (modeled using *C. striata* as a representative) is visualized. Red spheres highlight the four specific amino acid sites identified to be under positive selection in the ancestral lineage.

The aBSREL analysis uncovered a dichotomy in selective regimes between different lineages (Figure 4A, Table S2). The terminal branch leading to *C. pyrophthalmus* showed no evidence of positive selection (Mean *ω* ≈ 0.10), indicating a history of strong purifying selection that aligns with its specialized ecological niche. Conversely, we detected significant episodic positive selection (LRT, *p* = 0.022) at Node 5, which represents the ancestral lineage of the large-bodied clade (comprising *C. striata*, *C. marulius*, *C. argus*, etc.). On this ancestral branch, a small subset of sites (∼2.0%) in the *ND5* gene evolved under intense positive selection (ω = 116.2), driving the weighted mean ω to 2.40. This signal reflects episodic positive selection acting on a very small proportion of sites along this ancestral branch, while the majority of codons remained under strong purifying selection. Structural mapping of the four positively selected sites (209, 506, 521 and 550) onto the ND5 protein (Figure 4B) reveals that they are situated within the transmembrane domain and the lateral helix, pointing to potential functional adaptations in proton translocation or membrane interaction.

## 5. Structural Variation in the Control Region

**Figure 5:**
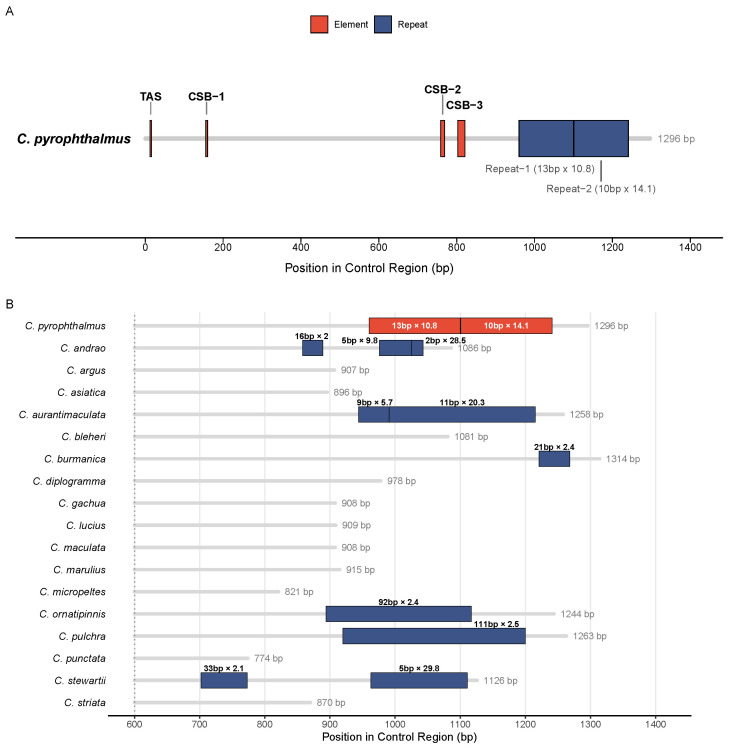
Structure and variation of the mitochondrial control region in *Channa*. (**A**) Schematic organization of the control region in *C. pyrophthalmus*. The linear map depicts the arrangement of conserved structural elements and tandem repeats. Red bars indicate conserved motifs: the TAS in the 5′ domain and conserved sequence blocks (CSB-1, CSB-2, and CSB-3) in the 3′ domain. Blue bars represent the two identified tandem repeat regions (Repeat-1 and Repeat-2). (**B**) Comparative analysis of tandem repeats across *Channa* species. The diagram illustrates the diversity of repeat organization within the variable 3′ domain (approx. 600–1400 bp) of the control region. Grey bars represent the full length of the control region for each species. Colored boxes denote the location and span of tandem repeats. Red boxes highlight the repeat regions in *C. pyrophthalmus*, while blue boxes represent those in other *Channa* species. Text labels above/below boxes indicate the repeat unit size (bp) × copy number. Non-integer copy numbers indicate the presence of partial or truncated repeat units at the boundaries of the tandem repeat region.

The control region of *C. pyrophthalmus* is characterized by standard conserved elements, including the termination-associated sequence (TAS) and three conserved sequence blocks (CSB-1, CSB-2, CSB-3) (Figure 5A). However, a broader comparison across the genus revealed significant structural plasticity in tandem repeats (Figure 5B). Specifically, *C. pyrophthalmus* contains two distinct sets of microsatellite-like tandem repeats within the variable 3′ domain. This architecture contrasts sharply with the extensive macro-repeats found in the *C. pulchra* and *C. ornatipinnis* lineages, highlighting the rapid evolutionary dynamics of this non-coding region.

## 6. Discussion

### 6.1. Phylogenetic Placement and Mitogenomic Conservatism in Channa

Resolving phylogenetic relationships within the genus *Channa* has historically been hindered by extensive morphological conservatism and phenotypic plasticity, particularly within species complexes such as the Gachua group and the Striata group [4,5]. Although single-locus mitochondrial markers like *COX1* and *CYTB* are widely used for rapid species identification, they often lack the resolution required to disentangle recent radiations and cryptic lineages [3,5,35]. By leveraging complete mitochondrial genomes, our study provides a robust phylogenetic framework that definitively positions *C. pyrophthalmus* as the sister lineage to *C. gachua* sensu stricto, with maximal nodal support across both maximum likelihood and Bayesian inference analyses. This result independently corroborates the recent morphological delimitation of *C. pyrophthalmus* and firmly embeds this taxon within the Gachua group [7,36].

Beyond resolving phylogenetic placement, our mitogenomic data clarify conflicting reports regarding structural rearrangements in *Channa* mitochondrial genomes. Previous studies proposed lineage-specific insertions between *tRNA-Met* and *ND2* as potential synapomorphies within the Gachua group [37]. However, the absence of such rearrangements in *C. pyrophthalmus* and in multiple reference mitogenomes examined here indicates that these features are likely homoplastic or population-specific events rather than clade-defining characters. Collectively, these findings highlight a high degree of structural conservatism in mitochondrial gene order across *Channa*, despite the remarkable ecological and morphological diversity of the genus [17,38].

### 6.2. Late Miocene Diversification and Indo-Burman Vicariance

Our time-calibrated phylogeny dates the divergence between *C. pyrophthalmus* and its sister lineage *C. gachua* to approximately 7.1 Ma. Geologially, the orogenesis of the Indo-Burman Ranges (IBR) is a protracted process; recent magnetostratigraphic studies indicate that the IBR had already emerged as a significant topographic barrier by the Early Miocene (∼23 Ma) [39,40].

The temporal lag between this initial uplift and the later biological divergence (∼7.1 Ma) suggests that ephemeral hydrological connectivity likely persisted across the IBR long after its initial rise. We propose that dynamic river capture events in the headwaters of the paleo-Brahmaputra and paleo-Irrawaddy systems maintained gene flow during the Mid-Miocene. Consequently, the intensification of crustal shortening and tectonic deformation in the Late Miocene—potentially coupled with the distinct geological dynamics of the southern IBR—constituted the decisive vicariant event. This tectonic reorganization finally severed these residual riverine connections, driving allopatric divergence [39,40,41].

Biogeographically, *C. pyrophthalmus* is confined to the Tanintharyi Region west of the Bilauktaung Range. Situated at the crossroads of the Indochinese and Sundaic faunas, this region likely functioned as a “coastal refugium” during regional tectonic upheaval [42]. The combined effects of mountain uplift and drainage isolation effectively encircled ancestral populations, fostering long-term genetic isolation and ultimately leading to the narrow endemism observed today. This pattern mirrors diversification processes documented in other freshwater taxa from the Indo-Burma region, underscoring the pivotal role of geological vicariance in shaping ichthyofaunal diversity [43].

### 6.3. Metabolic Evolution Underlying the Giant–Dwarf Dichotomy

A striking contrast in selective regimes emerges when comparing dwarf, range-restricted snakeheads with their large-bodied, widely distributed congeners. The terminal lineage leading to *C. pyrophthalmus* exhibits pervasive purifying selection across mitochondrial protein-coding genes, consistent with an evolutionary trajectory characterized by niche specialization and relative physiological stability. In contrast, the ancestral branch of the giant-bodied *Channa* clade shows evidence of episodic positive selection acting on the *ND5* gene, with a small subset of sites experiencing markedly elevated ω values.

*ND5* encodes a core subunit of mitochondrial Complex I and plays a central role in proton translocation and oxidative phosphorylation efficiency [44,45,46]. Structural mapping of positively selected sites indicates that positively selected substitutions are concentrated within transmembrane regions and lateral helices, where they may influence proton pumping mechanics or membrane interactions [47,48,49]. Similar patterns of adaptive evolution in *ND5* have been documented in teleost lineages facing high energetic demands, such as migratory salmonids and high-altitude fishes [50,51,52]. Taken together, these observations suggest that adaptive modifications of mitochondrial bioenergetics may have provided the physiological infrastructure for the evolution of large body size and active predatory lifestyles in giant snakeheads.

### 6.4. Lineage-Specific Acceleration of ATP8 and Cryptic Diversity

Among mitochondrial genes, *ATP8* displays the highest evolutionary rate across *Channa*, a pattern commonly attributed to relaxed functional constraints and short gene length. Notably, however, a lineage-specific signal of accelerated evolution was detected between *C. gachua* and *C. ornatipinnis*. Although estimates for short genes are inherently sensitive to stochastic effects [53], the restricted phylogenetic distribution of this signal suggests that it may reflect localized adaptive pressures rather than genome-wide relaxation.

The lineages exhibiting accelerated *ATP8* evolution predominantly inhabit fast-flowing hill streams, environments that impose sustained swimming demands and elevated metabolic turnover. Comparable signatures of *ATP8* adaptation have been reported in rheophilic and high-altitude teleosts, where modifications to ATP synthase are hypothesized to enhance energy production efficiency under challenging conditions [54,55,56,57]. Crucially, this finding resolves a discrepancy with a previous study [17], who reported a Ka/Ks ratio < 1.0 for *ATP8* in the same species comparison. We attribute this conflicting signal to the distinct phylogeographic origins of the reference sequences employed: Wang et al. utilized a lowland or introduced *C. gachua* from Guangzhou [16], whereas our analysis employed a native highland lineage from Yunnan [58]. This finding underscores the necessity of resolving the taxonomy of the *C. gachua* complex, as pooling distinct lineages obscures critical signals of adaptive evolution.

### 6.5. Structural Plasticity of the Mitochondrial Control Region

In contrast to the conserved architecture of coding regions, the mitochondrial control region emerges as a focal point of evolutionary dynamism within *Channa*. Comparative analyses reveal pronounced heterogeneity in control region length and repeat architecture, driven primarily by lineage-specific expansions and contractions of tandem repeat elements. *C. pyrophthalmus* possesses two distinct sets of microsatellite-like repeats within the variable 3′ domain, a configuration that differs markedly from the extensive macro-repeat arrays observed in species such as *C. pulchra* and *C. ornatipinnis*.

This structural plasticity is consistent with replication slippage as a major mechanism shaping control region evolution in teleost mitogenomes [59]. Importantly, the species-specific nature of these repeat motifs suggests that non-coding regions of the mitochondrial genome evolve under relaxed selective constraints, accumulating variation at a pace far exceeding that of protein-coding genes. As such, the control region represents a promising molecular marker for resolving shallow phylogeographic structure and population differentiation within *Channa*, particularly in species complexes where coding regions provide limited resolution [60,61].

### 6.6. Methodological Considerations and Data Limitations

While mitochondrial genomes provide powerful insights into phylogenetic relationships and lineage-specific evolutionary processes, several methodological considerations warrant attention. This study is based on a single mitochondrial genome of *C. pyrophthalmus*, which limits the assessment of intraspecific variation. As a maternally inherited locus, mitochondrial DNA captures only a subset of the species’ evolutionary history, and potential mitonuclear discordance cannot be ruled out without nuclear markers [62]. Furthermore, given the narrow endemic status of *C. pyrophthalmus*, future studies integrating nuclear genomics would be valuable not only for corroborating these evolutionary patterns but also for assessing genetic diversity to inform conservation strategies. Given that *C. pyrophthalmus* is a narrow-range endemic targeted by the international aquarium trade, establishing such genetic baselines is urgent for monitoring potential overexploitation [63].

In addition, divergence time estimates rely on a secondary calibration point due to the paucity of confidently assignable internal fossils within Channidae. Although this approach provides a reasonable temporal framework for comparative inference, absolute age estimates should be treated with caution [64]. Importantly, the evolutionary interpretations advanced in this study are grounded primarily in relative divergence patterns and their congruence with regional geological events. Future research integrating broader geographic sampling and genome-wide nuclear data will be essential for testing the robustness of these hypotheses and for advancing a more comprehensive understanding of snakehead evolution [65].

## 7. Conclusions

By generating the first complete mitochondrial genome of *C. pyrophthalmus*, this study resolves the phylogenetic placement of a recently described, geographically restricted snakehead and firmly embeds it within the Gachua group as the sister lineage to *C. gachua* sensu stricto. Comparative mitogenomic analyses reveal a conserved coding architecture contrasted by pronounced structural plasticity in the mitochondrial control region, underscoring its potential as a marker for resolving shallow evolutionary patterns.

Integrating time-calibrated phylogenetic inference with selection analyses, our results suggest that Late Miocene geological vicariance in the Indo-Burman region and lineage-specific shifts in mitochondrial bioenergetics jointly contributed to diversification within the genus. The contrasting selective regimes between dwarf and giant snakeheads highlight a potential link between mitochondrial metabolic evolution, body size divergence, and ecological specialization. Together, these findings provide a comprehensive framework for understanding diversification of freshwater snakeheads and establish a foundation for future population-level and nuclear genomic investigations.

## Figures and Tables

**Table 1:** Annotation of the mitochondrial genome of *C. pyrophthalmus*.

Gene	Position		Length (bp)	Intergenic Nucleotides	Code		Strand	Anticodon
	From	To			Start	Stop		
*tRNA-Phe*	1	68	68	0			H	GAA
*12S rRNA*	69	1016	948	0			H	
*tRNA-Val*	1017	1089	73	0			H	TAC
*16S rRNA*	1090	2772	1683	0			H	
*tRNA-Leu(UUR)*	2773	2843	71	0			H	TAA
*ND1*	2844	3818	975	3	ATG	TAA	H	
*tRNA-Ile*	3823	3892	70	4			H	GAT
*tRNA-Gln*	3892	3962	71	−1			L	TTG
*tRNA-Met*	3962	4031	70	−1			H	CAT
*ND2*	4032	5078	1047	0	ATG	TAA	H	
*tRNA-Trp*	5079	5147	69	−1			H	TCA
*tRNA-Ala*	5149	5217	69	1			L	TGC
*tRNA-Asn*	5219	5291	73	1			L	GTT
*tRNA-Cys*	5329	5393	65	37			L	GCA
*tRNA-Tyr*	5394	5463	70	0			L	GTA
*COX1*	5465	7006	1542	1	GTG	TAA	H	
*tRNA-Ser(UCN)*	7016	7086	71	9			L	TGA
*tRNA-Asp*	7090	7161	72	3			H	GTC
*COX2*	7169	7859	691	7	ATG	T	H	
*tRNA-Lys*	7860	7934	75	0			H	TTT
*ATP8*	7936	8103	168	1	ATG	TAA	H	
*ATP6*	8094	8777	684	−10	ATG	TAA	H	
*COX3*	8777	9562	786	−1	ATG	TAA	H	
*tRNA-Gly*	9562	9630	69	−1			H	TCC
*ND3*	9631	9979	349	0	ATA	T	H	
*tRNA-Arg*	9980	10,047	68	0			H	TCG
*ND4L*	10,048	10,344	297	0	ATG	TAA	H	
*ND4*	10,338	11,718	1381	−7	ATG	T	H	
*tRNA-His*	11,719	11,787	69	0			H	GTG
*tRNA-Ser(AGY)*	11,788	11,855	68	0			H	GCT
*tRNA-Leu(CUN)*	11,858	11,930	73	2			H	TAG
*ND5*	11,931	13,763	1833	0	ATG	TAA	H	
*ND6*	13,760	14,281	522	−4	ATG	TAA	L	
*tRNA-Glu*	14,282	14,350	69	0			L	TTC
*CYTB*	14,355	15,495	1141	4	ATG	T	H	
*tRNA-Thr*	15,496	15,568	73	0			H	TGT
*tRNA-Pro*	15,569	15,638	70	−1			L	TGG
Control Region	15,637	16,632	996	0			H	

## Data Availability

The DNA sequences generated and analyzed during the current study are available in the GenBank repository under accession number PX764270.

## Supplementary Materials

The following supporting information can be downloaded at: https://www.mdpi.com/article/10.3390/ani16030467/s1, Figure S1: Molecular authentication of the studied specimen using mitochondrial *COX1* sequences; Figure S2: Relative Synonymous Codon Usage and amino acid composition of the *Channa pyrophthalmus* mitochondrial genome; Figure S3: Putative secondary structures of the 22 transfer RNAs (tRNAs) in the *Channa pyrophthalmus* mitochondrial genome; Figure S4: Substitution saturation analysis of the concatenated dataset of 13 mitochondrial protein-coding genes; Figure S5: Phylogenetic tree of the genus *Channa* based on 13 mitochondrial protein-coding genes; Table S1: List of mitochondrial genomes of *Channa* species used for comparative and phylogenetic analyses; Table S2: Results of the adaptive Branch-Site Random Effects Likelihood (aBSREL) analysis testing for episodic positive selection on mitochondrial protein-coding genes.
