# Supplementary material for: Comparative Mitogenomics of Channa pyrophthalmus Unveils Orogeny-Driven Speciation and Lineage-Specific Adaptive Evolution in Snakeheads
Source: Animals (Basel). 2026 Feb 2;16(3):467. doi: 10.3390/ani16030467 (PMC12896699; doi:10.3390/ani16030467)
Supplement: Supplementary file 1 [file animals-16-00467-s001.zip › Figure S1 Molecular authentication of the studied specimen using mitochondrial COX1 sequences.pdf]

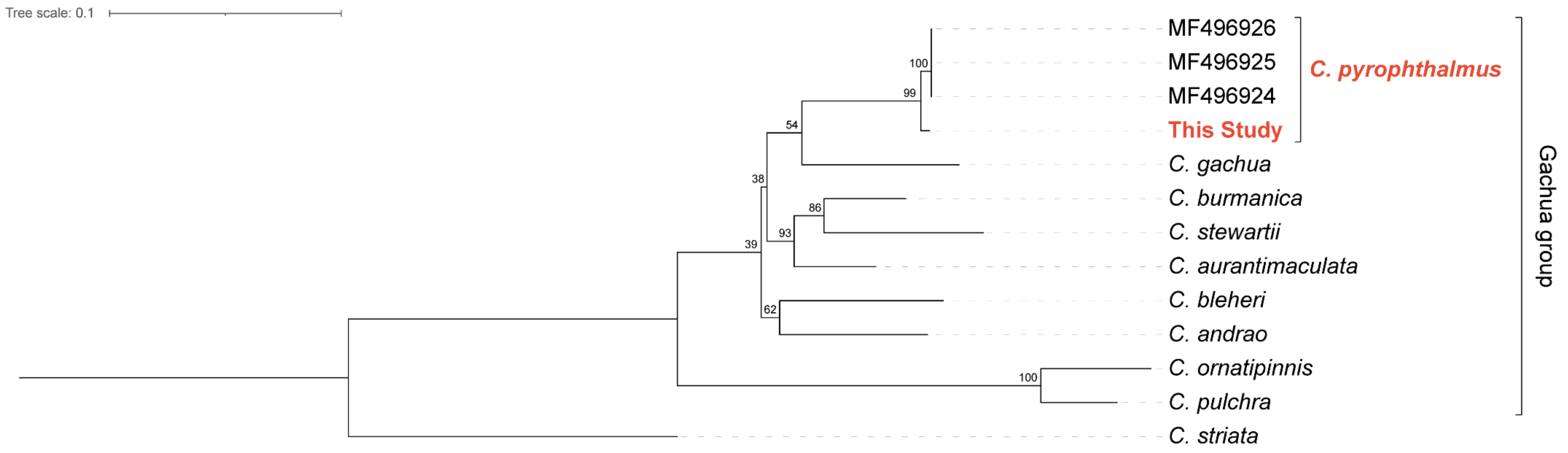

Figure S1 Molecular authentication of the studied specimen using mitochondrial *COX1* sequences. The phylogenetic tree was constructed using the Maximum Likelihood (ML) method. The specimen sequenced in this study forms a strongly supported monophyletic clade with the reference sequences of *Channa pyrophthalmus* (GenBank Accession Nos. MF496924–MF496926), distinct from the sister taxon *C. gachua*. Numbers at nodes indicate bootstrap support values.
