# Supplementary material for: Comparative Mitogenomics of Channa pyrophthalmus Unveils Orogeny-Driven Speciation and Lineage-Specific Adaptive Evolution in Snakeheads
Source: Animals (Basel). 2026 Feb 2;16(3):467. doi: 10.3390/ani16030467 (PMC12896699; doi:10.3390/ani16030467)
Supplement: Supplementary file 1 [file animals-16-00467-s001.zip › Figure S2 Relative Synonymous Codon Usage and amino acid composition of the Channa pyrophthalmus mitochondrial genome.pdf]

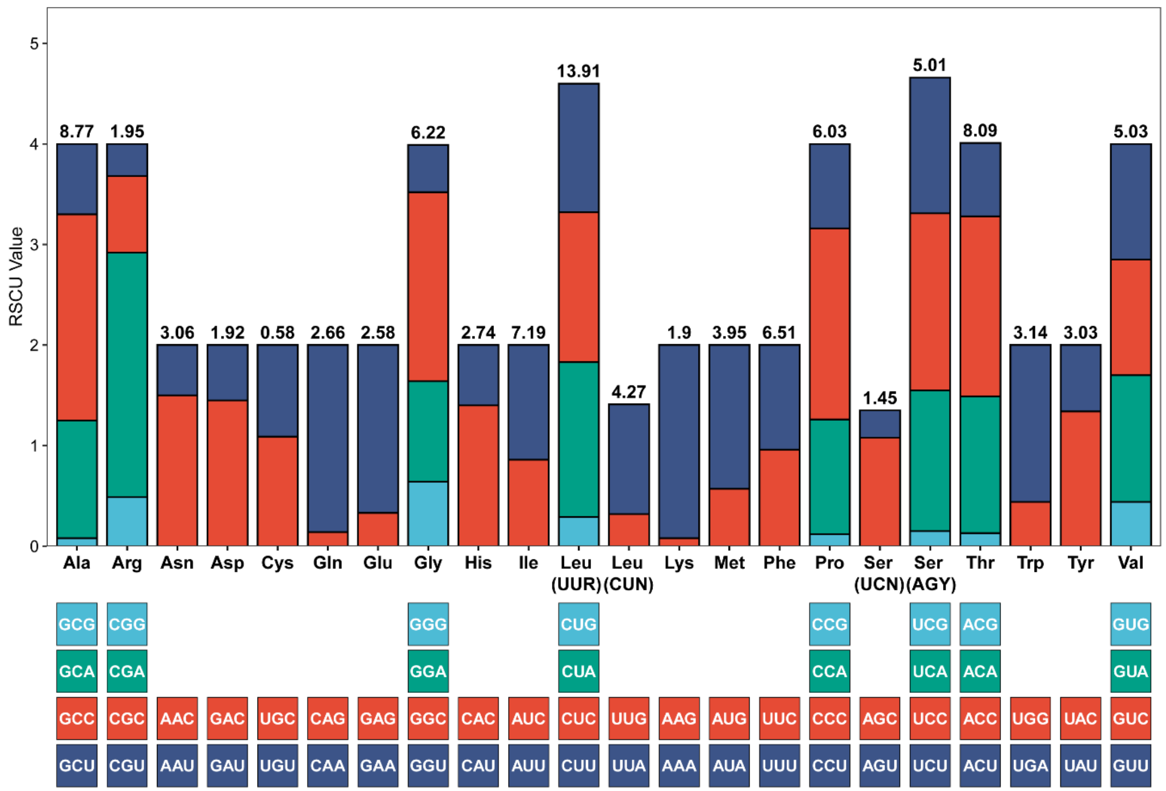

Figure S2. Relative Synonymous Codon Usage (RSCU) and amino acid composition of the *Channa pyrophthalmus* mitochondrial genome. The analysis includes all 13 mitochondrial protein-coding genes. The x-axis displays the amino acid families, with Leucine (Leu) and Serine (Ser) separated into two groups based on their codon families (UUR/CUN and UCN/AGY). The y-axis represents the cumulative RSCU values, where the height of each colored segment corresponds to the RSCU value of a specific codon. The specific codons corresponding to the colored segments are listed at the bottom of the chart. The numbers above each column indicate the percentage frequency of that amino acid in the total mitochondrial protein sequences.
