# Supplementary material for: Comparative Mitogenomics of Channa pyrophthalmus Unveils Orogeny-Driven Speciation and Lineage-Specific Adaptive Evolution in Snakeheads
Source: Animals (Basel). 2026 Feb 2;16(3):467. doi: 10.3390/ani16030467 (PMC12896699; doi:10.3390/ani16030467)
Supplement: Supplementary file 1 [file animals-16-00467-s001.zip › Figure S3 Putative secondary structures of the 22 transfer RNAs (tRNAs) in the Channa pyrophthalmus mitochondrial genome.pdf]

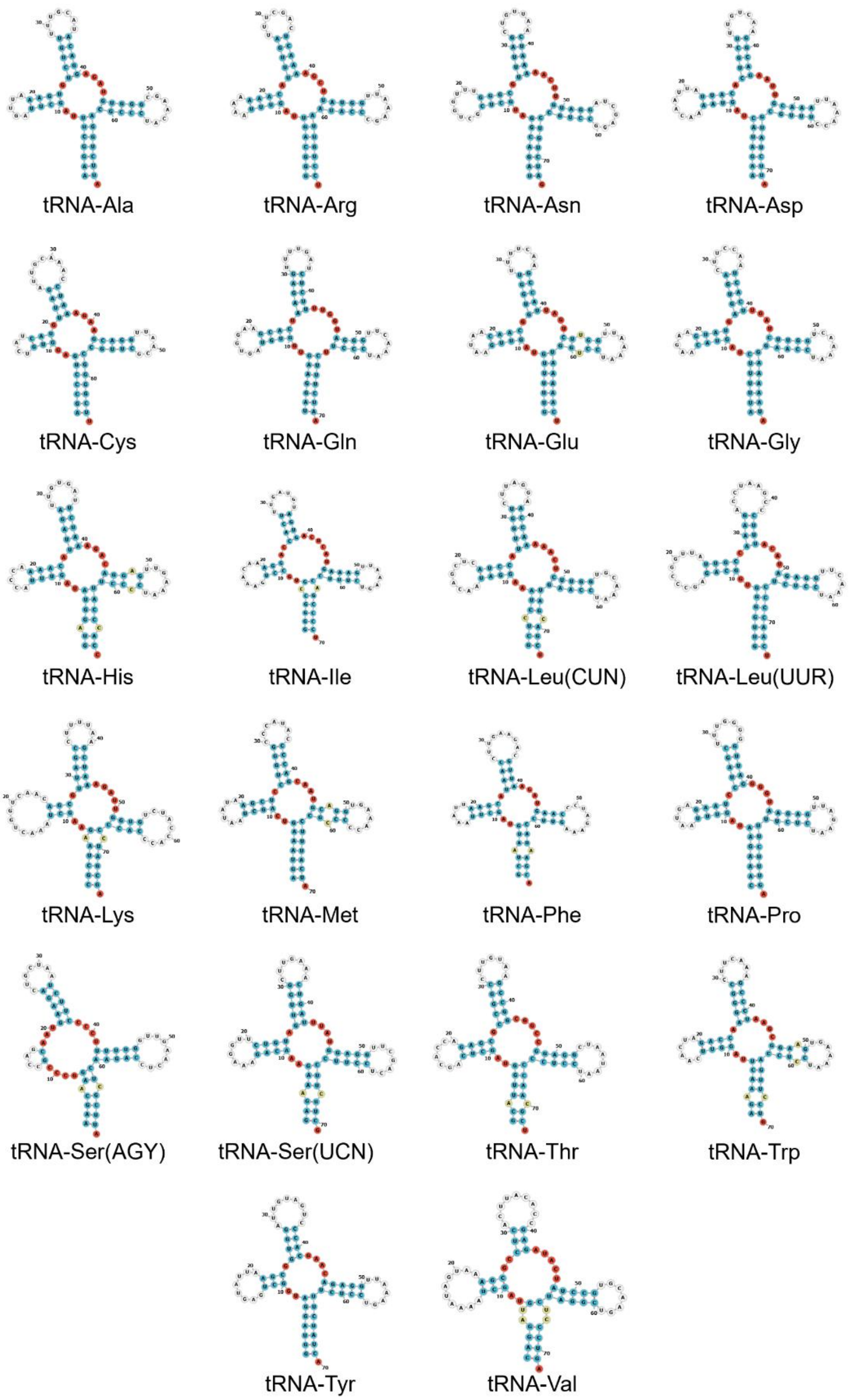

Figure S3. Putative secondary structures of the 22 transfer RNAs (tRNAs) in the *Channa pyrophthalmus* mitochondrial genome. The nucleotide sequences are arranged to show the folding patterns. All tRNAs fold into the canonical cloverleaf structure with one exception. tRNA-Ser (AGY) lacks the DHU arm.
