# Supplementary material for: Comparative Mitogenomics of Channa pyrophthalmus Unveils Orogeny-Driven Speciation and Lineage-Specific Adaptive Evolution in Snakeheads
Source: Animals (Basel). 2026 Feb 2;16(3):467. doi: 10.3390/ani16030467 (PMC12896699; doi:10.3390/ani16030467)
Supplement: Supplementary file 1 [file animals-16-00467-s001.zip › Figure S4 Substitution saturation analysis of the concatenated dataset of 13 mitochondrial protein-coding genes.pdf]

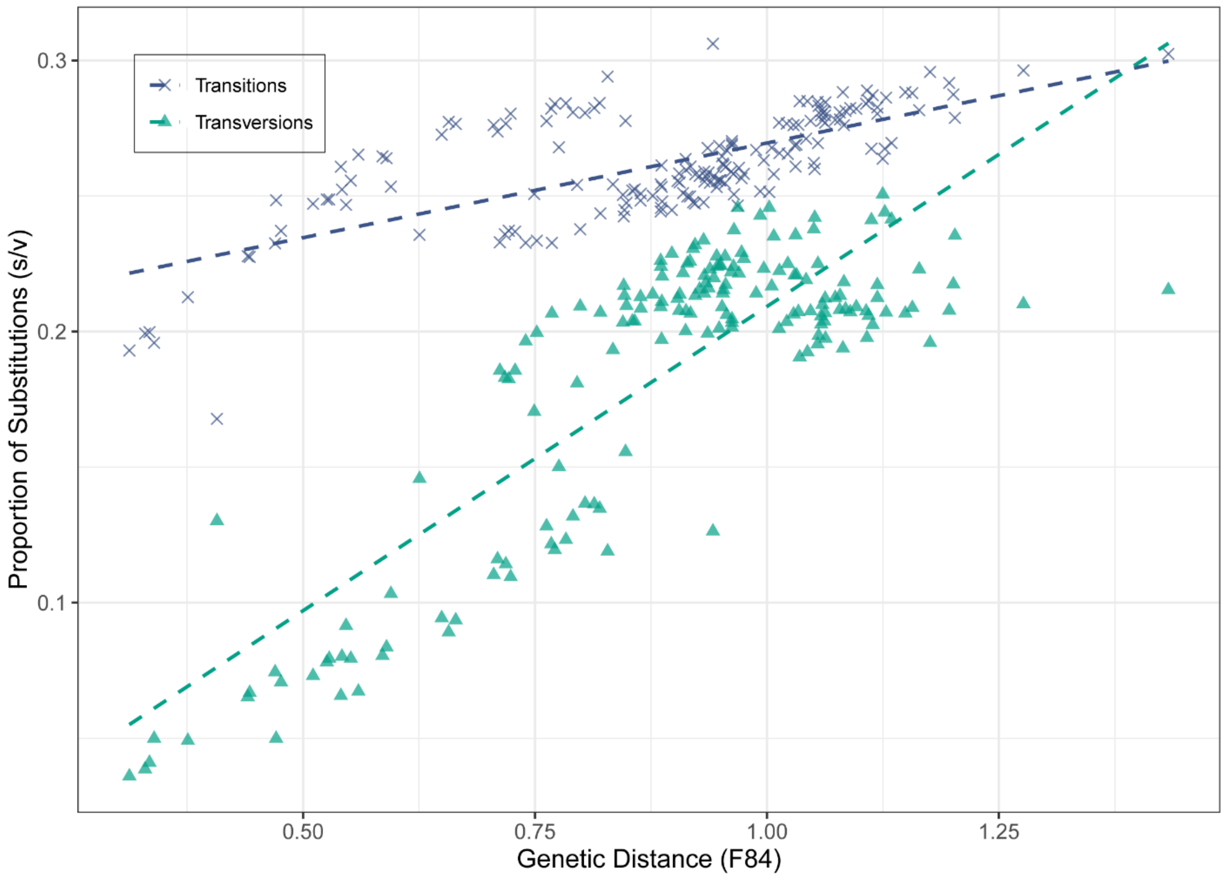

Figure S4. Substitution saturation plot for the 13 mitochondrial protein-coding genes. Transitions (blue crosses) and transversions (green triangles) are plotted against F84 genetic distance. The linear increase in both substitution types indicates that no substitution saturation occurred in the dataset.
