# Supplementary material for: Comparative Mitogenomics of Channa pyrophthalmus Unveils Orogeny-Driven Speciation and Lineage-Specific Adaptive Evolution in Snakeheads
Source: Animals (Basel). 2026 Feb 2;16(3):467. doi: 10.3390/ani16030467 (PMC12896699; doi:10.3390/ani16030467)
Supplement: Supplementary file 1 [file animals-16-00467-s001.zip › Figure S5 Phylogenetic tree of the genus Channa based on 13 mitochondrial protein-coding genes.pdf]

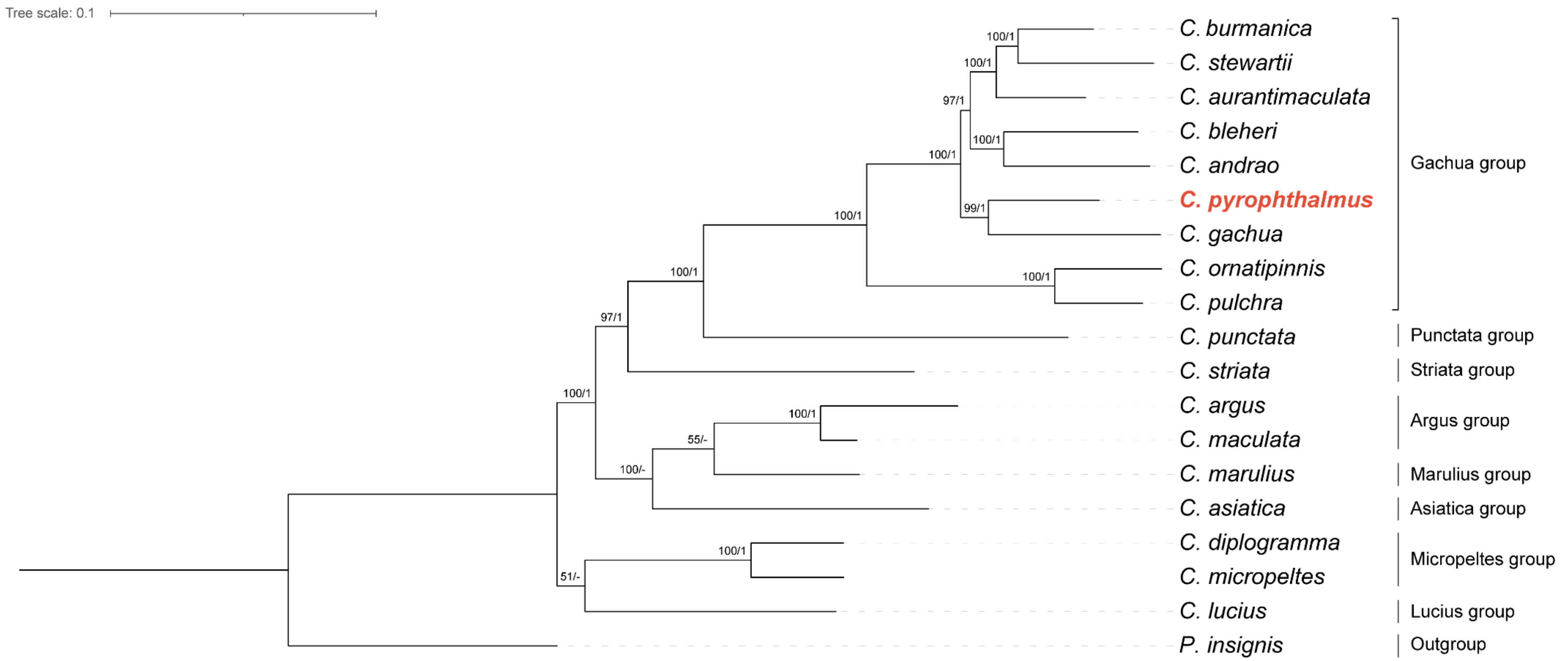


**Figure S5. Phylogenetic tree of the genus *Channa* based on 13 mitochondrial protein-coding genes.** The topology was inferred using both Maximum Likelihood (ML) and Bayesian Inference (BI) methods. Nodal numbers indicate ML bootstrap support percentages and Bayesian posterior probabilities, respectively (BS / PP). The focal species, *Channa pyrophthalmus*, is highlighted in red.
