# Supplementary material for: Comparative Mitogenomics of Channa pyrophthalmus Unveils Orogeny-Driven Speciation and Lineage-Specific Adaptive Evolution in Snakeheads
Source: Animals (Basel). 2026 Feb 2;16(3):467. doi: 10.3390/ani16030467 (PMC12896699; doi:10.3390/ani16030467)
Supplement: Supplementary file 1 [file animals-16-00467-s001.zip › Table S1 List of mitochondrial genomes of Channa species used for comparative and phylogenetic analyses.pdf]

Table S1. Information on the mitochondrial genomes, native distribution, and maximum body size of the *Channa* species analyzed.

Species	GenBank Accession No.	Length (bp)	Native Distribution	Max length (cm)*
Channa pyrophthalmus	PX764270	16,932	Myanmar (Tanintharyi Region, west of Bilauktaung Range)	12.7
Channa andrao	NC_071938	16,729	India (Brahmaputra basin)	14.2
Channa argus	KC823605	16,558	East Asia (China, Korea, Russia)	150.0
Channa asiatica	NC_025225	16,550	Southern China & Northern Vietnam	23.5
Channa aurantimaculata	NC_072726	16,897	India (Brahmaputra basin)	36.2
Channa bleheri	NC_082225	16,714	India (Brahmaputra basin)	14.4
Channa burmanica	NC_072609	16,953	Myanmar (Irrawaddy basin)	8.0
Channa diplogramma	MG986721	16,571	Southern India (Western Ghats)	44.0
Channa gachua	NC_036948	16,547	Widespread (South to Southeast Asia)	32.9
Channa lucius	MF804538	16,570	Southeast Asia (Indochina to Sundaland)	53.0
Channa maculata	KC823606	16,559	Southern China & Northern Vietnam	60.0
Channa marulius	NC_022713	16,569	Widespread (South to Southeast Asia)	183.0
Channa micropeltes	NC_030542	16,567	Southeast Asia (Indochina to Sundaland)	130.0
Channa ornatipinnis	NC_071894	16,886	Myanmar (Rakhine State)	20.5
Channa pulchra	NC_071893	16,895	Myanmar (Rakhine State)	30.0
Channa punctata	NC_042213	16,409	Widespread (Indian Subcontinent)	31.0
Channa stewartii	NC_071939	16,765	Eastern Himalaya (India and Nepal)	29.7
Channa striata	NC_032037	16,509	Widespread (South to East Asia)	100.0
Parachanna insignis	NC_022480	16,607	Central Africa (Congo basin)	53.2

* Maximum standard lengths were retrieved from FishBase (https://www.fishbase.se/home.htm) and original species descriptions.
