# Supplementary material for: Comparative Mitogenomics of Channa pyrophthalmus Unveils Orogeny-Driven Speciation and Lineage-Specific Adaptive Evolution in Snakeheads
Source: Animals (Basel). 2026 Feb 2;16(3):467. doi: 10.3390/ani16030467 (PMC12896699; doi:10.3390/ani16030467)
Supplement: Supplementary file 1 [file animals-16-00467-s001.zip › Table S2. Results of the adaptive Branch-Site Random Effects Likelihood (aBSREL) analysis testing for episodic positive selection on mitochondrial protein-coding genes.pdf]

Table S2. Results of the adaptive Branch-Site Random Effects Likelihood (aBSREL) analysis testing for episodic positive selection on mitochondrial protein-coding genes.

Gene	Branch	LRT	Corrected P-value	Mean ω	Rate Class 1 (ω1 / %)	Rate Class 2 (ω2 / %)	Significance
ND5	Node5 (*C. striata* + Node6)	12.51	0.02	2.40	0.04 / 98.0	116.22 / 2.0	Yes
ND5	*C. andrao*	0.07	1	0.06	0.03 / 97.6	1.50 / 2.3	No
ND5	*C. argus*	0	1	0.10	0 / 87.5	0.79 / 12.5	No
ND5	*C. asiatica*	0.76	1	0.09	0.01 / 96.6	2.17 / 3.4	No
ND5	*C. aurantimaculata*	0	1	0.05	0.05 / 100	-	No
ND5	*C. bleheri*	0	1	0.07	0.07 / 100	-	No
ND5	*C. burmanica*	0	1	0.08	0.08 / 100	-	No
ND5	*C. diplogramma*	0	1	0.05	0.05 / 100	-	No
ND5	*C. gachua*	0	1	0.07	0.07 / 100	-	No
ND5	*C. lucius*	0	1	0.10	0 / 86.6	-	No
ND5	*C. maculata*	0	1	0.08	0.08 / 100	-	No
ND5	*C. marulius*	0	1	0.05	0.05 / 100	-	No
ND5	*C. micropeltes*	3.36	1	11.65	0.02 / 99.2	1376.19 / 0.1	No
ND5	*C. ornatipinnis*	0	1	0.08	0.08 / 100	-	No
ND5	*C. pulchra*	0	1	0.04	0.04 / 100	-	No
ND5	*C. punctata*	0.36	1	0.10	0.04 / 97.2	2.35 / 2.8	No
ND5	*C. pyrophthalmus*	0	1	0.06	0.06 / 100	-	No
ND5	*C. stewartii*	0.79	1	0.08	0.04 / 99.0	4.07 / 1.0	No
ND5	*C. striata*	0	1	0.07	0 / 89.5	0.65 / 10.5	No
ND5	Node10 (*C. maculata* + *C. marulius*)	0	1	0.20	0.20 / 100	-	No
ND5	Node14 (*C. lucius* + Node15)	0	1	0.40	0.40 / 100	-	No
ND5	Node15 (*C.micropeltes* + *C. diplogramma*)	3.05	1	0.20	0 / 93.3	2.98 / 6.7	No
ND5	Node2 (Node3 + Node24)	0	1	0.02	0.02 / 100	-	No
ND5	Node21 (*C. ornatipinnis* + *C. pulchra*)	0.33	1	0.19	0.05 / 94.0	2.43 / 6.0	No

Gene	Branch	LRT	Corrected P-value	Mean ω	Rate Class 1 ($\omega 1$ / %)	Rate Class 2 ($\omega 2$ / %)	Significance
ND5	Node24 (Node25 + Node30)	0	1	0.00	0.00 / 100	-	No
ND5	Node25 (*C. aurantimaculata* + Node27)	0	1	0.02	0.02 /100	-	No
ND5	Node27 (*C. burmanica* + *C. stewartii*)	0	1	0.04	0.04 / 100	-	No
ND5	Node3 (Node4 + Node21)	4.65	1	0.28	0 / 93.6	4.34 / 6.4	No
ND5	Node30 (*C. gachua* + *C. pyrophthalmus* clade)	1.72	1	0.21	0 / 99.4	37.43 / 1.0	No
ND5	Node4 (*C. punctata* + Node5)	1.34	1	0.19	0.04 / 99.2	18.5 / 1.0	No
ND5	Node6 (Node7 + Node14)	0	1	0.03	0.03 / 100	-	No
ND5	Node7 (*C. asiatica* + Node8)	0.43	1	0.13	0.06 / 98.8	5.74 / 1.2	No
ND5	Node8 (*C. argus* + Node10)	0	1	0.05	0.05 / 100	-	No
ATP6	*C. pyrophthalmus*	0	1	0.10	0.10 / 100	-	No
ATP8	*C. pyrophthalmus*	0	1	0.66	0.66 / 100	-	No
COX1	*C. pyrophthalmus*	0	1	0.01	0.01 / 100	-	No
COX2	*C. pyrophthalmus*	0	1	0.03	0.03 / 100	-	No
COX3	*C. pyrophthalmus*	0	1	0.02	0.02 / 100	-	No
CTYB	*C. pyrophthalmus*	0.20	1	0.06	0.01 / 97.8	2.17 / 2.2	No
ND1	*C. pyrophthalmus*	0	1	0.04	0.04 / 100	-	No
ND2	*C. pyrophthalmus*	0	1	0.06	0.06 / 100	-	No
ND3	*C. pyrophthalmus*	0	1	0.08	0.08 / 100	-	No
ND4	*C. pyrophthalmus*	0.42	1	0.10	0.04 / 98.8	5.11 / 1.2	No
ND4L	*C. pyrophthalmus*	0	1	0.04	0.04 / 100	-	No
ND6	*C. pyrophthalmus*	0	1	0.06	0.06 / 100	-	No
